# Free Radical Exposure Creates Paler Carotenoid-Based Ornaments: A Possible Interaction in the Expression of Black and Red Traits

**DOI:** 10.1371/journal.pone.0019403

**Published:** 2011-04-27

**Authors:** Carlos Alonso-Alvarez, Ismael Galván

**Affiliations:** 1 Ecology Unit, Instituto de Investigación en Recursos Cinegéticos, IREC (CSIC, UCLM, JCCM), Ciudad Real, Spain; 2 Department of Evolutionary Ecology, Museo Nacional de Ciencias Naturales (CSIC), Madrid, Spain; University of Debrecen, Hungary

## Abstract

Oxidative stress could be a key selective force shaping the expression of colored traits produced by the primary animal pigments in integuments: carotenoids and melanins. However, the impact of oxidative stress on melanic ornaments has only recently been explored, whereas its role in the expression of carotenoid-based traits is not fully understood. An interesting study case is that of those animal species simultaneously expressing both kinds of ornaments, such as the red-legged partridge (*Alectoris rufa*). In this bird, individuals exposed to an exogenous source of free radicals (diquat) during their development produced larger eumelanin-based (black) plumage traits than controls. Here, we show that the same red-legged partridges exposed to diquat simultaneously developed paler carotenoid-based ornaments (red beak and eye rings), and carried lower circulating carotenoid levels as well as lower levels of some lipids involved in carotenoid transport in the bloodstream (i.e., cholesterol). Moreover, partridges treated with a hormone that stimulates eumelanin production (i.e., alpha-melanocyte-stimulating hormone) also increased blood carotenoid levels, but this effect was not mirrored in the expression of carotenoid-based traits. The redness of carotenoid-based ornaments and the size of a conspicuous eumelanic trait (the black bib) were negatively correlated in control birds, suggesting a physiological trade-off during development. These findings contradict recent studies questioning the sensitivity of carotenoids to oxidative stress. Nonetheless, the impact of free radicals on plasma carotenoids seems to be partially mediated by changes in cholesterol metabolism, and not by direct carotenoid destruction/consumption. The results highlight the capacity of oxidative stress to create multiple phenotypes during development through differential effects on carotenoids and melanins, raising questions about evolutionary constraints involved in the production of multiple ornaments by the same organism.

## Introduction

Oxidative stress is the imbalance between the rate of production of reactive oxygen and nitrogen species (ROS and RNS; including free radicals) by the cell metabolism and the state of the repair and antioxidant machinery [1]. Oxidative stress is intimately involved in key evolutionary trade-offs between growth, reproduction and survival (self-maintenance), as accelerated growth and reproductive functions require metabolic changes, which may unbalance the pro-oxidant-antioxidant equilibrium [2], [3]. Oxidative stress should thus have an important role in the design of phenotypes including those traits used in animal communication (i.e., signals) [4].

It is well known that many colored patches of animal integuments are produced by carotenoid pigments, which have antioxidant properties [5]. In vertebrates, the antioxidant properties of carotenoids and the fact that they cannot be synthesized and have to be ingested with food imply that carotenoid-based traits should be costly to produce, and hence, may reliably signal the quality of the bearer [6], [7]. Carotenoids acquired in the diet should be optimally distributed among competing functions, that is, allocated to either signal expression or to reduce oxidative stress [7]. However, the hypothesis that individuals exposed to high oxidative stress sacrifice their investment in signaling has only been tested once by directly manipulating ROS/RNS levels under *in vivo* conditions: Isaksson & Andersson [8] increased ROS levels by administering a pro-oxidant compound in drinking water (i.e., paraquat) to great tits (*Parus major*). In contrast to expectations, they did not detect any decline in the expression of a carotenoid-based plumage ornament nor in the levels of circulating carotenoids, results that seem to support recent arguments that question the relevance of carotenoids as antioxidants [9], [10].

On the other hand, melanins are the most common pigments in vertebrates, producing many yellow-brownish (pheomelanin) and grey-black (eumelanin) traits [11]. Melanins are synthesized by the organisms, suggesting that melanin-based ornaments may not be costly to produce (e.g., [11], [12]). Moreover, the expression of melanic traits is tightly controlled by genes [13], [14], supporting the view that they may not have evolved as reliable signals of individual quality [12]. Thus, it is often assumed that the information content of carotenoid- and melanin-based signals differs, e.g., [15], [16]. However, the artificial decrease of the blood levels of an antioxidant involved in melanin synthesis (i.e., glutathione) led to increase the expression of eumelanin-based black plumage in developing great tits [17] and adult greenfinches (*Carduelis chloris*) [18]. High glutathione levels blocks the production of eumelanin in melanocytes [19], which led us to propose that eumelanin-based traits could act as handicap signals (*sensu* Zahavi 1975) [20] because they would reveal the capacity of individuals to face the antioxidant challenge imposed by melanin synthesis [17]. Accordingly, those great tits forced to endure low glutathione levels increased the levels of plasmatic antioxidants [17], whereas greenfinches showed a higher oxidative damage [18]. Pheomelanic ornaments, in contrast, could directly indicate glutathione levels of an individual because pheomelanin synthesis requires glutathione as a substrate [19].

Therefore, if we assume that oxidative stress can shape the expression of both carotenoid- and melanin-based traits, what is expected when both kinds of traits are simultaneously expressed in the same organism? Signaling theory proposes different hypotheses to explain the evolution of multiple ornaments [21], [22]. The “multiple messages” hypothesis posits that each signal provides information about a different aspect of quality, whereas the redundant (“back-up”) hypothesis suggests that every signal provides an overall measure of quality, all signals being used to reduce errors in the receiver's assessment [23], [24]. The possibility that both carotenoid- and melanin-based traits reveal the antioxidant status of the bearer would support the second hypothesis.

**Figure 1:**
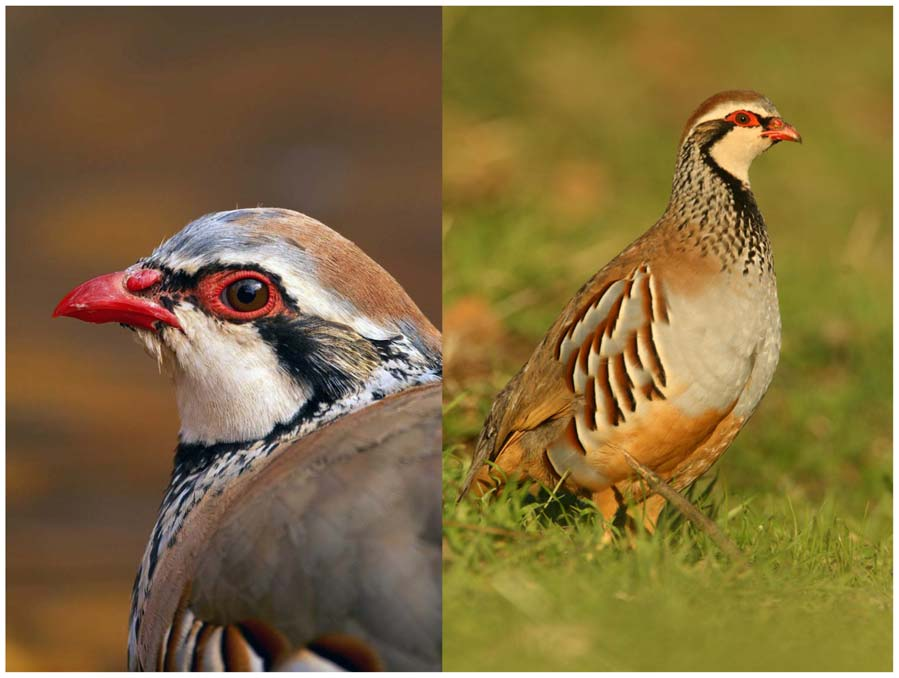
The red-legged partridge. The red carotenoid-based traits of the head (bill and eye rings; left) and the black spotted bib and flank bands (eumelanin) and red-brownish flank bands (pheomelanin) of the plumage (right) are shown.

Here we test the hypothesis that a trade-off exists between allocating carotenoids to combat oxidative stress and to signaling functions. In particular we test the prediction that both circulating carotenoid levels and the amount of carotenoids allocated to the ornaments decrease with high oxidative stress. Additionally, we hypothesize that the expression of both carotenoid- and melanin-based traits in the same individual should be equally influenced by oxidative stress. If oxidative stress decreases the availability of all antioxidant resources (i.e., including circulating carotenoids and intracellular glutathione), the expression of carotenoid- and pheomelanin-based traits should be inhibited, but the expression of eumelanin-based traits should in turn be enhanced under high oxidative stress. To test our predictions, oxidative stress and α-melanocyte stimulating hormone (α-MSH; a hormone that stimulates eumelanin production [25]) levels were manipulated during development in red-legged partridges (*Alectoris rufa*), a species that displays both carotenoid and melanin-based traits (Fig. 1). We have recently tested a fully independent set of hypotheses for the same experiment [26] (Text S1), and found that partridges exposed to exogenous ROS decreased glutathione levels, produced larger eumelanin-based traits (black bib and flank bands) and developed smaller pheomelanin-based traits (brown flank bands) in their plumage than controls [26]. Meanwhile, partridges treated with α-MSH showed the same results regarding the expression of melanin-based traits, but without any change in antioxidant levels [26]. Therefore, we predict here that the treatment with α-MSH does not have an effect on carotenoid levels in plasma nor in the expression of carotenoid-based traits, but that the exogenous source of ROS decreases circulating carotenoid levels and the expression of carotenoid-based traits (i.e., beak and eye-ring redness).

## Materials and Methods

### Ethics statement

The work met the Spanish legal requirements about animal welfare (approval identification: PAI06-0018; Consejería de Medio Ambiente, Junta de Comunidades de Castilla La Mancha; JCCM) and was supervised by the veterinary staff of Instituto de Investigación en Recursos Cinegéticos (Ciudad Real, Spain).

### Study model

Red-legged partridges show conspicuous traits (Fig. 1) with a slight sexual dichromatism (males are redder) [27]. Red pigmentation in the head is produced by carotenoids and is positively correlated to cell-mediated immune response and size-corrected body mass (i.e., “body condition”) [28], [29]. It is also sensitive to food restriction [29] and predicts resistance to oxidative damage after an experimental immune challenge [30]. Female partridges doubled their reproductive investment (i.e., clutch size) when the redness of their mates is experimentally intensified (C Alonso-Alvarez, unpublished). Positive correlations between the shape of the spotted black bib, cell-mediated immunity and body condition have also been detected (L Pérez-Rodríguez and FR Mougeot, unpublished), whereas the size of black bands have been positively related to body condition in males and negatively to the heterophil:lymphocyte ratio in females (a physiological stress index), suggesting that they may act as a signals of quality in both sexes [31].

### Experimental design

Seventy-seven partridges were obtained from eggs laid by 25 breeding pairs housed in outdoor cages at the *Dehesa Galiana* experimental facility (Ciudad Real, Spain) [26]. Cages were inspected daily, and the eggs removed, identified and immediately stored at 15°C, which arrested embryo development. Stored eggs were transferred to incubators every 15 days. Hatchlings from two successive incubation series were used (hatching dates: 8 and 23 June, 2008). Hatchlings were identified with plastic rings and kept in indoor aviaries with *ad libitum* food and water. Birds were randomly assigned to one of the four treatments (below) just before the development of their melanized plumage (i.e., about 20-days of age). Then, they were transferred to one of four different indoor aviaries (4×3×3 m; Ligth:Dark; 13 h∶11 h; *ad libitum* food and water). Diet was pelleted food (Perdix Vuelo, Superfeed, Madrid) and wheat (1∶1; see Text S1). Birds from the second incubation series were kept in four other aviaries under the same conditions. Birds received diquat (*n* = 22), α-MSH (*n* = 17) or both (*n* = 17), while a fourth group served as a control (*n* = 21). Treatments were balanced with regard to parents' identity, thus aiming to represent all treatments in each brood.

Just before the chicks were assigned to the treatments and placed in the aviaries (20 days of age), they were sampled for blood, weighed and their tarsus length measured with a caliper (“pre-treatment values”). The same was done at the end of the manipulation, i.e., 33 days later (“post-treatment values”). Blood was stored at 4°C and centrifuged within 5 h after extraction. Plasma and cell fractions were stored at −80°C. We introduced the hour of blood sampling as a covariate in models testing lipid peroxidation, triglycerides or cholesterol levels (see Statistical analyses below and Text S1).

### Diquat treatment

Diquat dibromide is a molecule similar to paraquat that generates superoxide anion, one of the most abundant ROS, e.g., [32], [33]. The commercial product ‘Reglone’ (Syngenta, Madrid), which consisted of 20% w/v of diquat dibromide in water, was used. In our study, diquat was supplied in drinking water. The treatment with diquat or only water lasted 33 days, overlapping with the greater part of feather development. Each aviary contained a tank with treated water (Text S1). Therefore, each aviary contained a single drinking treatment (i.e., diquat plus water or only water).

The diquat dose (0.50 ml l^−1^ in water) was established on the basis of a previous pilot study (Text S1). Taking into account water intake during that study, mean daily intake of diquat-treated birds was ≅ 7 µg of diquat. Body mass and tarsus length did not significantly differ between treatments neither before nor after the treatment [26]. Body mass gain and final body condition were not affected [26]. Changes in behavior and feces (diarrhea or hemorrhage) were daily inspected, but nothing was detected. No bird died during the pilot study or the subsequent manipulation, though some birds died before the color assessment (40 days after the end of the diquat treatment; below).

### α-MSH treatment

α-MSH (Sigma, St. Louis, MO) was injected in the left pectoral muscle of each experimental bird (0.04 mgs of α-MSH in 2 ml of phosphate buffered saline; PBS). Control birds received 2 ml of PBS only. Injections were administered every two days during a 15 day period (i.e., 8 injections per bird). Each bird received a total amount of 0·32 mg of α-MSH. The total α-MSH dose used in this study is equivalent to that used by Höhn & Braun [34] (also Text S1). Both α-MSH- and PBS-injected birds were present in each aviary.

### Molecular sexing

Blood cell pellet was used. DNA from sex chromosomes was amplified by PCR (primers 2550F and 2718R) [35].

### Assessment of circulating carotenoid, cholesterol and triglycerides

Total plasma carotenoid levels were determined by spectrophotometry (Shimadzu UV-1603, Japan), using a standard curve of lutein (Sigma, St Louis, MO) (see Text S1).

Plasma levels of total cholesterol and triglycerides (two of the main lipoprotein components) were also assessed by spectrophotometry (A25, Biosystems SA, Barcelona). The cholesterol oxidase/peroxidase and glycerol phosphate oxidase/peroxidase methods were used to determine them, respectively (commercial kits; Biosystems SA, Barcelona, see Text S1). Lipoproteins serve as carotenoid carriers, and particularly, plasma cholesterol levels would indicate levels of those lipoproteins carrying carotenoids as they have been positively correlated with circulating carotenoid levels in birds, e.g., [5], [36]. Meanwhile, plasma triglycerides were assessed as a proxy of changes in intestinal absorption because these lipids are the main constituents of those lipoproteins (i.e., chylomicrons) in charge of lipid and carotenoid absorption from intestines to blood (about 93% of chylomicron mass) [37].

### Color assessment of carotenoid-based traits

Digital photography (Olympus E-500 camera) was used after checking the relatively low repeatability of different spectrometers, probably due to the heterogeneous surface of partridge bare parts (see Text S1). Previous studies in the same species have shown that carotenoid-based color as assessed in the present study is associated with fitness-related variables [28]–[30], [38], [39]. We assume that technical limitations could have reduced the precision of the measure, but not its accuracy. Thus significant effects detected from our measurements would be revealed in spite of this limitation, but not due to methodological bias. See Text S1 for additional details and support for this technique.

Partridges were photographed at 93 days of life, when all the birds showed a fully developed adult phenotype. Three diquat-treated, two α-MSH-treated and one control bird died between the end of the treatment and this age (*χ* ^2^ = 3.12, d.f. = 3, *P* = 0.373). Thus, the color of seventy-one birds was finally assessed. Birds were held in the same posture and at a fixed distance from the camera by means of a repro lighting unit. To correct for subtle variability in the level of light received by the partridges, a standard red chip (Kodak) was placed close to the red traits of the bird (Text S1 for further details).

Hue values were obtained from mean RGB using the algorithm described in Foley & Van Dam [40]. A principal components analysis (PCA) was carried out to obtain a single variable from the three measures of redness. A single component explained 91.9% of variance. Eigenvalues were 0.930, 0.954, 0.874 for upper and lower mandible and eye ring, respectively. The three variables were positively correlated among them (all Pearson's *r*-values *>*0.82, all *P*'*s*<0.001). As in the case of hue, high PC1 values denoted less redness. However, to facilitate the interpretation, the sign of the PC1 values was reversed. This new variable was termed as “red intensity”.

### Assessment of melanin-based plumage

The expression of the melanin-based plumage was also analyzed from digital pictures and Adobe Photoshop at the same age. These pictures were different that those used for the redness assessment. The surfaces of black spotted bib and black and brown flank bands (pixels) were obtained (Text S1 and ref. [26]).

### Statistical analyses

Generalized linear mixed models (PROC MIXED, SAS software) [41] were used to determine the experimental effects on red intensity and change in the level of circulating carotenoids and lipids (i.e., post-treatment minus pre-treatment value). These variables were dependent ones in single GLMMs, and diquat (presence vs. absence) treatment, α-MSH (presence vs. absence) treatment and sex were added as factors together with their interactions. The identity the pair that laid the egg and the aviary in which the chicks were reared were included as random factors. In the model testing red intensity, the hue of the red chip was added as a covariate (*F*_1,57.7_ = 145.7, *P*<0.001). The pre-treatment value was included as a covariate in models testing blood parameters, e.g., [39]. To understand whether effects were mediated by body mass variability (i.e., potential differences in food intake or metabolism), body mass change was also added as a covariate. This covariate was the residual of the difference between post- and pre-treatment masses (dependent) corrected by the pre-treatment mass (independent). Lastly, the time of blood sampling was tested as a covariate to control for any influence of the last feeding event on lipid values (always *p*>0.20, and hence, removed).

Starting with the saturated model, a backward stepwise procedure was used, removing any term with *P*>0.05. Random factors were maintained in the models, though similar results were obtained when removed. Non-significant terms included in significant interactions were also maintained. The Satterthwaite correction was used to approximate the degrees of freedom. The distribution of residuals obtained from the models met the normality assumption. Only the results of the models obtained after the stepwise procedure are shown here. Full saturated models are reported in Text S1. Least squares means and slopes (± s.e.) were calculated from the models.

## Results

### Effects of diquat and α-MSH on the expression of a carotenoid-based trait

**Figure 2:**
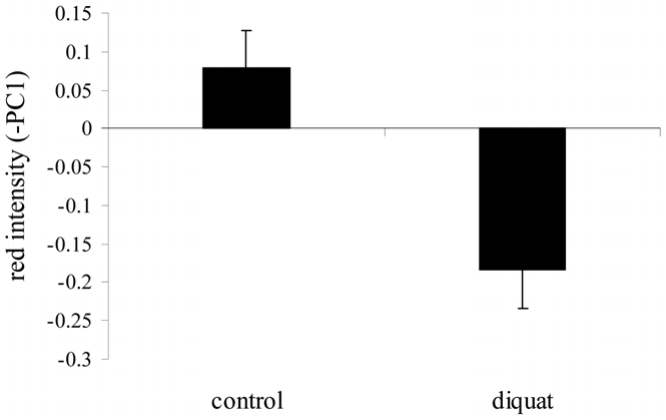
Redness of carotenoid-based ornaments of control partridges and partridges exposed to free radicals. Red intensity is the inverse of a principal component summarizing variability in the hue of both mandibles and eye ring (methods). Least square means ± s.e. were obtained from the final model (results).

Partridges exposed to diquat during development showed paler red traits than controls (Fig. 2; diquat treatment: *F*_1,61_ = 5.63, *P* = 0.020; red reference chip *F*_1,57.7_ = 145.7, *P*<0.001). The α-MSH-treatment, sex and interactions did not show significant effects, being removed from the final best fitted model (all *P's*>0.16; saturated models in Text S1).

### Effects of diquat and α-MSH on circulating carotenoids and lipids

**Figure 3:**
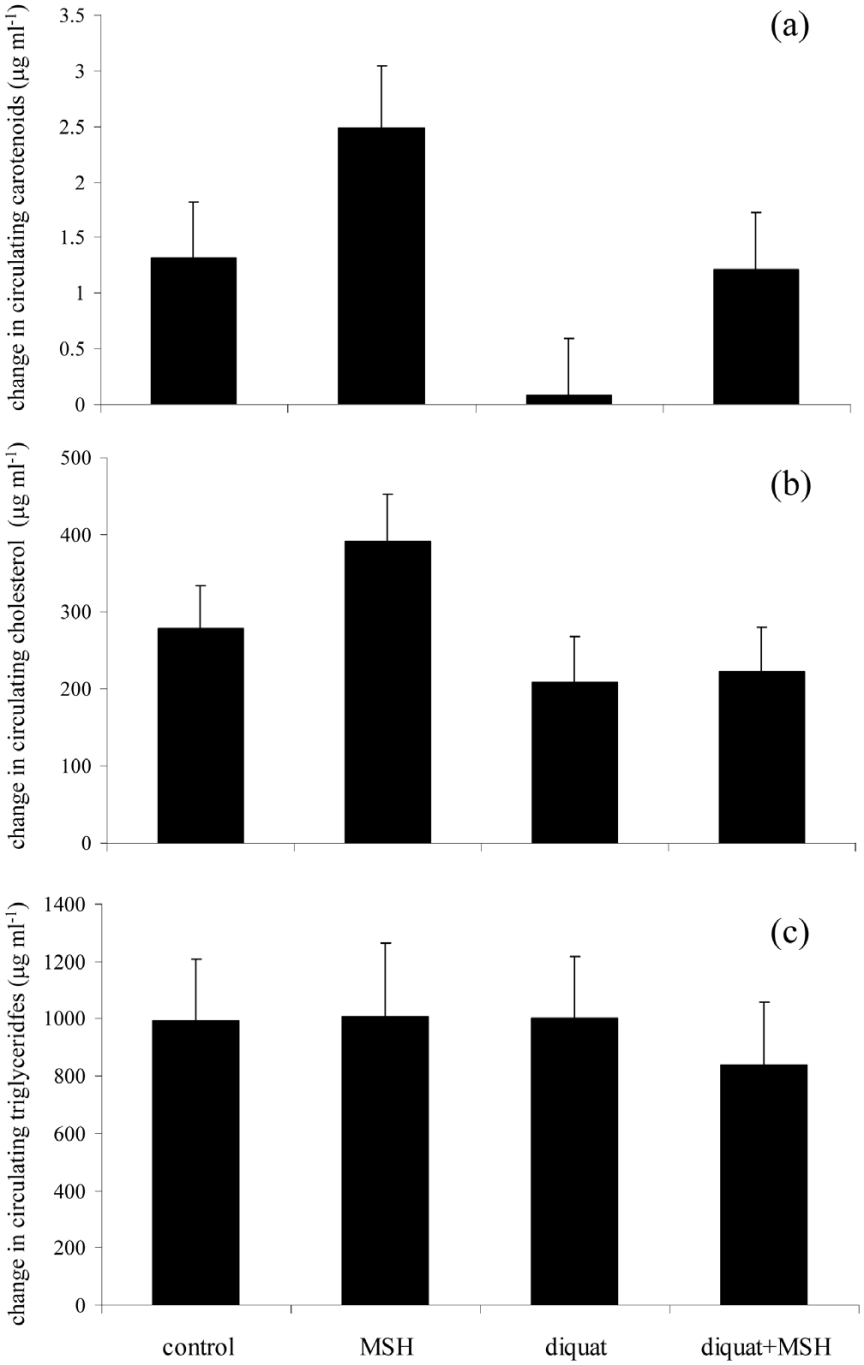
Change in the levels of several blood parameters during the experiment. Circulating (a) carotenoids, (b) cholesterol and (c) triglycerides in partridges exposed to different treatments during development. Least square means ± s.e. were obtained from the final models (results).

In the model analyzing variability in carotenoid levels, both diquat (*F*_1,38.1_ = 4.73, *P* = 0.036) and α-MSH (*F*_1,59_ = 6.03, *P* = 0.017) treatments had significant effects. Partridges exposed to diquat showed a weaker increase in carotenoid levels than control birds (0.59±0.46 and 1.81±0.47 µg ml^−1^, respectively). In contrast, birds treated with α-MSH showed a greater carotenoid increase than controls (1.79±0.45 and 0.61±0.44 µg ml^−1^, respectively). Body mass change (*F*_1,48.7_ = 12.94, *P* = 0.001; slope: +0.041±0.011) and pre-treatment carotenoid level (*F*_1,26.8_ = 69.2, *P*<0.001; slope: -0.776±0.093) remained in the model. For illustrative purposes, figure 3 represents the change in carotenoid levels separately for each experimental group. Nonetheless, the diquat x α-MSH interaction was not significant (*P* = 0.908).

Partridges exposed to diquat also showed a weaker increase in cholesterol levels than controls (+168.2±63.2 and 315.3±59.4 µg ml^−1^, respectively; *F*_1,46_ = 4.75, *P* = 0.034; pre-treatment level: *F*_1,56.5_ = 28.71, *P*<0.001, slope: −0.716±0.140). α-MSH, sex, body mass gain and interactions did not reveal any significant influence on cholesterol variability (all *P*'s>0.19), and were removed from the model (also Fig. 3b). By contrast, the change in triglyceride levels was only explained by body mass change (*F*_1,50.9_ = 4.10, *P* = 0.048, slope: 9.232±4.558) and pre-treatment value (*F*_1,60.7_ = 57.9, *P*<0.001, slope: −0.925±0.122; other terms: *P*'s>0.10; Fig. 3c).

Interestingly, if the change in cholesterol is added as a covariate to the final model for change in carotenoid levels (above; cholesterol change: *F*_1,56.4_ = 5.06, *P* = 0.028, slope: +0.028±0.013), both diquat and α-MSH factors became non-significant (*P* = 0.157 and 0.141, respectively). However, the model for carotenoid levels was not affected by the change in triglyceride levels (always *P*>0.35).

### Carotenoid- and melanin-based trait expressions interact under oxidative stress

**Figure 4:**
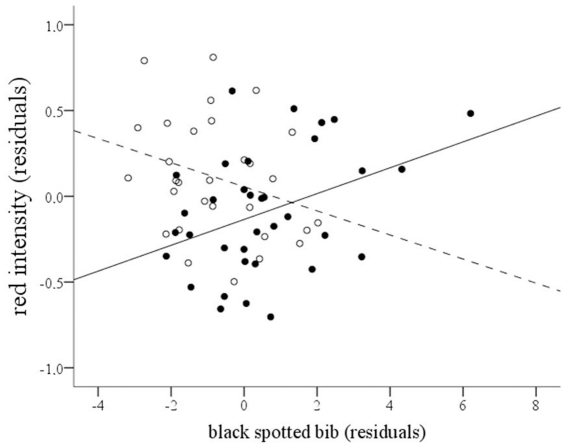
Relationship between red intensity and size of the black spotted bib. Partridges exposed to diquat (full dots and continuous line) and control birds (empty dots and spotted line). Red intensity values are residuals after controlling for red chip variability and random factors. Bib data are residuals after controlling for the effect of α-MSH-treatment and random factors (all *P*'s<0.05). The lines were calculated from the model (see slopes in Results).

We explored the influence of the variability in the expression of melanic traits on the expression of carotenoid-based traits. With this aim, we added the surface of black (eumelanic) spotted bib, black (eumelanic) and brown (pheomelanic) flank bands as covariates to the model for red color intensity (see above). Black and brown flank bands did not show any significant influence or interaction with any factor, and were removed from the model (all *P*'s>0.06). However, the surface of the black spotted bib interacted with diquat-treatment (*F*_1,58.4_ = 8.12, *P* = 0.006; Fig. 4). Red intensity was positively related to the area of the bib in diquat-treated birds (slope  =  +0.127±0.040, *P* = 0.004), and negatively related in birds not exposed to the pro-oxidant (slope = −0.110±0.062), though non-significantly different from zero (*P* = 0.17). If the bird with the largest bib is conservatively removed from the analyses (see Fig. 4), both the interaction and the relationship between redness and bib surface in diquat-treated birds remained significant (*P* = 0.015 and 0.022, respectively). Furthermore, if the analysis is restricted to birds that did not receive any treatment (*n* = 17), the negative relationship between bib surface and redness is significant (*F*_1,15_ = 10.23, *P* = 0.006).

## Discussion

These results support our prediction that individuals exposed to high ROS levels should reduce the allocation of carotenoids to the integument, not only producing paler ornaments, but also reducing plasma carotenoid levels. On the other hand, our results, in addition to those reported in a recent article from the same experiment (i.e., [26]) are the first to show that a single stressor is able to simultaneously shape the expression of both carotenoid- and melanin-based traits in the same species, e.g., [15], [18], [42]. Partridges exposed to an exogenous source of ROS during development not only showed a reduced expression of carotenoid- and pheomelanin-based traits, but also larger eumelanic (black) plumage patches than controls [26]. In contrast to its stimulating effect on eumelanin [26], α-MSH did not affect the expression of carotenoid-based traits, but induced an increase in plasma carotenoid levels. This finding might be related to the pleiotropic effects of this hormone, which is involved in many organismal functions whose activity may demand carotenoids (e.g., immunity) [14], [25].

The information content of carotenoid-based signals has been much debated during recent years. Hartley and Kennedy [9] proposed that carotenoid allocation to signaling could compromise physiological functions other than antioxidant protection (e.g., vision, gene activation, immunity), or alternatively, that carotenoid-dependent traits could indicate the availability of other antioxidants that would protect carotenoids from being bleached by ROS. Correlational studies and those where carotenoid availability was experimentally increased did not support a strong negative link between carotenoid and oxidative stress levels, at least among avian species [10], [43], [44]. However, only Isaksson and Andersson [8] have addressed the problem by manipulating the exposure to ROS under *in vivo* conditions to assess simultaneously its impact on circulating carotenoid levels and carotenoid-based ornaments. They did not detect any significant effect on both traits in adult great tits exposed to paraquat. Recently, Hõrak et al. [18] induced high oxidative damage levels by chemically inhibiting the synthesis of glutathione in adult greenfinches, which led to an increase in the expression of eumelanic feathers, but not in the expression of carotenoid-based feathers. Our results contrast with these findings. We can argue that such a disparity may be due to differences between the experimental procedures (e.g., growing vs. adult animals) or perhaps due to species- or tissue-specific responses (i.e., plumage vs. bare parts), which underlines the difficulties inherent to accept any null hypothesis.

Nonetheless, the fact that the weak increase in plasma carotenoid levels in diquat-treated birds was correlated with changes in circulating cholesterol levels suggests that oxidative stress did not directly impact on carotenoid molecules. We could argue that our findings were the result of an impairment of the capacity to absorb lipids and carotenoids due to diquat-induced damage on intestines. However, no significant effect was detected on body mass gain or body condition, which should be expected when lipid absorption is disrupted. Furthermore, no signs of diarrhea or intestinal hemorrhage were detected, and changes in triglyceride levels were not parallel to cholesterol changes. In fact, triglycerides constitute the most abundant components of lipoproteins that carry lipids and carotenoids from the intestine to the bloodstream (i.e., chylomicrons) [37], [45], [46]. Instead, the results suggest that high ROS levels could have specifically altered the cholesterol metabolism by blocking cholesterol synthesis. Indeed, pro-oxidant diquat-like molecules oxidize cholesterol, generating oxysterols [47], [48] that, in turn, inhibit cholesterol synthesis (reviewed in ref. [49]). This inhibitory effect could have ultimately limited the amount of circulating carotenoids.

Our results also reveal that the expression of carotenoid- and melanin-based ornaments in the same individual may not be independent. The possibility that the production of both kinds of ornaments rely on the same organismal resources (i.e., antioxidants) challenges the assumption that the information content of carotenoid- and melanin-based signals qualitatively differ [12] and has implications for understanding how multiple ornaments are integrated in developmental processes [50]. The fact that both traits could simultaneously signal the state of the antioxidant machinery also supports the hypothesis that multiple ornaments act as redundant (“back-up”) signals [23]. It is assumed that redundant traits evolve because the receiver of the signals (or cues) better perceives a general message [51]. Nonetheless, we should also consider that the information conferred by carotenoid- and melanin-based traits could be used by different receivers [52]. Carotenoid-based ornaments have usually linked to intersexual selection, whereas melanin-based signals seem mostly act as status signals in intra-sexual competition [12]. We must also take into account that the properties of the integuments where melanin and carotenoids are deposited substantially differ in red-legged partridges. Feather composition remains stable until the next molt, whereas carotenoids in bare parts can be very sensitive to rapid changes in physical condition [38], [53]. This scenario may favor the existence of temporal trade-offs in the production of both traits, for instance, by increasing the allocation of carotenoids to bare parts when feathers have been fully developed. Along the same lines, the negative correlation in the expression of red head traits and black bib detected in non-manipulated birds suggests that producing both types of ornaments at the same level could be particularly costly, e.g., [52], [54], [55]. We must emphasize that this is a correlative result and future experimental work is required. Nonetheless, the finding supports the hypothesis proposed by Hebets & Papaj [22] suggesting that a signaler's ability to generate a signal constrain its ability to generate a second signal, making a simultaneous generation of both traits difficult and hence potentially useful to assess the signaler's quality.

To conclude, our results highlight the potential role of oxidative stress in the developmental plasticity of phenotypes [56]. The level of exposure to exogenous and/or endogenous sources of oxidative stress throughout development may broadly vary among individuals. Maternal effects in the form of differential allocation of antioxidants (carotenoids, vitamins) [57] or pro-oxidant molecules (testosterone) [58] to the egg yolk could determine the individual capacity to face oxidative challenges during life. Furthermore, environmental changes may accelerate growth, which could increase metabolic demands, ultimately leading to a higher oxidative stress [59], [60]. Lastly, in addition to endogenous ROS due to cell respiration, environmental stressors such as some pollutants, ultraviolet-light or radiation [2], [61] could act during development, promoting oxidative stress and shaping the expression of signals. Further studies under free-living conditions are now required to fully demonstrate the oxidative-stress-based link between carotenoid- and melanin-based ornaments.

## Supporting Information

Text S1 Supporting information.
(DOC)
