# Supplementary material for: Free Radical Exposure Creates Paler Carotenoid-Based Ornaments: A Possible Interaction in the Expression of Black and Red Traits
Source: PLoS One. 2011 Apr 27;6(4):e19403. doi: 10.1371/journal.pone.0019403 (PMC3083443; doi:10.1371/journal.pone.0019403)
Supplement: Text S1 — Supporting information. (DOC) [file pone.0019403.s001.doc]

**Supporting Information**

**The material published in Galvan & Alonso-Alvarez (2009)**

We want to underline that the material published in Galvan & Alonso-Alvarez (2009; Proc R Soc B 276: 3089–3097) is substantially different that the material included in the present manuscript. Galvan & Alonso-Alvarez (2009)’s study addressed a fully independent set of hypotheses with different implications. It was not possible to publish all the material in a single article because addressing all the hypotheses would have implied surpassing the length limits of any conventional journal (the cited article in fact reached the limits for Proc R Soc B). Data on carotenoid-dependent color variability, plasma carotenoid levels as well as cholesterol and triglyceride levels were not analyzed in Galvan & Alonso-Alvarez (2009). Moreover, interactions between melanin- and carotenoid-based color expressions were not analyzed in the other article.

**Some sampling details**

At the time of blood sampling all the birds were simultaneously removed from the aviaries and stored in cages. Then birds were randomly taken by a person blind to the bird’s identity. Thus, the time between the last feeding event and blood sampling should mostly depend on the time of bleeding.

**Diquat treatment and pilot study**

Diquat was administered in one tank (4 liters) per aviary. Water was replaced every 4 days to avoid the disintegration of the diquat molecule (i.e., following product properties).

To serve as an orientation for the diquat dose, a pilot study was previously performed on another group of 20-day old partridges (*n* = 36). These birds were randomly assigned to one of four doses of diquat (0.25, 0.50, 1 and 2 ml diquat per litre of water; *n* = 7 birds per group), or received water only (*n* = 8). These sample sizes were chosen due to our ethical concern regarding the consequences of diquat administration (we must remember that diquat is a toxic compound). Each group was kept in a separate aviary (conditions described above). Body masses were recorded at the beginning and at the end of the study (15-day interval). A blood sample was also taken at the end of the study. Blood was centrifuged within 5h from extraction. Red blood cell fraction was stored at -85ºC. The amount of lipid peroxidation in erythrocytes was determined following the procedure described in Galvan & Alonso-Alvarez (2009 Proc R Soc B 276: 3089–3097). To know whether water intake was influenced by diquat, daily water consumption was estimated during four days. A Spearman correlation test showed that diquat doses were unrelated to water intake (*r*_s_ = -0.53, *n* = 5, *P* = 0.36). Mean values were: 16, 14, 14, 16 and 11 ml of water in control, 0.25-, 0.50-, 1- and 2-ml/L-groups, respectively. The intermediate dose (i.e. 0.50 ml/L) was chosen for use in the definitive experiment on the basis of the body mass variability (Supporting Fig. 1a). Results from lipid peroxidation analyses supported this choice (Supporting Fig. 1b).

**Supporting Figure 1. Pilot study.** Body mass change (a) as the residuals of the regression between change in body mass (post- minus pre-treatment masses) and pre-treatment mass, and (b) amount of lipid peroxidation in erythrocytes at the end of the study. Means ± s.e.

**α-MSH treatment**

α-MSH dose was calculated on the basis of Höhn & Braun (1980)’s study (i.e., Auk 97: 601–607) about Ptarmigans (genus *Lagopus*), but considering differences in body mass between both species. We must however be cautious in interpreting our α-MSH-induced effects as we did not know variability in α-MSH levels in red-legged partridges under free-living conditions.

**Diet composition**

Birds were fed with 50% food mass of wheat and 50% of commercial pelleted food (Perdix Vuelo, Superfeed, Madrid). Wheat contains carotenoids, i.e. lutein, zeaxanthin and beta-carotene, in order of abundance from major to minor (e.g. Moore et al. 2005 J Agric Food Chem 53: 6649-6657). Pelleted food contained protein 20.3%, fat 4.5%, Methionine 0.45%, cellulose 3.7% and ash 7.3%. It had also several additives: vit A 15000 IU/Kg, vit D3 3000 IU/Kg, vit E 22.5mg/Kg, Cu 10.5mg/Kg.

**Quantification of total carotenoids in plasma**

Plasma (60 μl) was diluted in acetone (1:10) and mixed, and the flocculent protein precipitated by centrifuging at 11000 g for 10 min. The absorbance of the supernatant was determined at 446 nm. Carotenoid values assessed twice on a sub-sample were highly repeatable (*r* = 0.97, *P* < 0.001, *n* = 25; following Lessells & Boag 1987, Auk 104: 116–121, here and thereafter). One post-treatment and two pre-treatment samples from three different birds could not be assessed due to lack of plasma volume.

**Quantification of cholesterol and tryglicerydes in plasma**

The absorbance of samples (10 μl each technique) was measured at 505 nm (A25-Autoanalyzer, Biosystems SA, Barcelona). Both techniques assessed twice on a subsample were highly repeatable (both: *r* > 0.95, *P* < 0.001, *n* = 30). In both parameters, one post-treatment and four pre-treatment samples from different birds could not be assessed due to lack of volume.

**Measurement of redness by means of digital photography**

In order to assess color in red carotenoid-based ornaments of red-legged partridges, we previously checked three different methods: a traditional full-wavelength spectrometer (Avantes DH-2000, Eerbeek, The Netherlands; 300-700 nm), a portable spectrometer (Minolta 2600d, Japan; 360-700 nm) and digital photography (Olympus E-500, Japan) with a repro lighting unit (Repro Base with lights RB260 2x11W 6000ºK; Kaiser Fototechnik, Buchen). We decided to use the last one because the surfaces of upper and lower mandibles and eye rings are very irregular (rounded in the beak and very thin in the eye ring; see fig. 1 of the main text) and the probe of both spectrometers did not adapt well to them, allowing the entrance of light. We, however, acknowledge that spectrometers may be a very useful tool when used on plumage as they adapt well to its surface. In partridge’s bare parts, the repeatability of the Avantes DH-2000 measurements in a single point was low (triplicates in upper mandible and eye ring showed *r* < 0.6) probably due to its heterogeneous surface. The probe tip of most 300-700 nm spectrometers assesses about 1-2 mm^2^ of surface, though it can illuminate a disc of about 3 mm^2^. However, the red surfaces in our partridges are quite large and heterogeneous (about 4-5 cm^2^ in the picture). This would have required performing a very large number of spectrometer measurements, taking into account the low repeatability of each measurement that would require triplicate every one. Instead, by using digital software we assessed the redness of the complete red surface, which probably captures most part of color variation, though sacrificing UV detection.

However, the contribution of UV light in carotenoid-based traits of red-legged partridges is relatively low. The figure below (Supporting Figure 2) shows the variability in the full reflectance spectrum in the eye ring of red-legged partridges from a previous work. Please see how the amplitude in the error bars is reduced in the UV part of the spectrum (300-400nm). A figure showing the reflectance spectrum for the upper mandible can also be found in Perez-Rodriguez 2008 Behav Ecol Sociobiol 62: 995–1005 (p. 998).


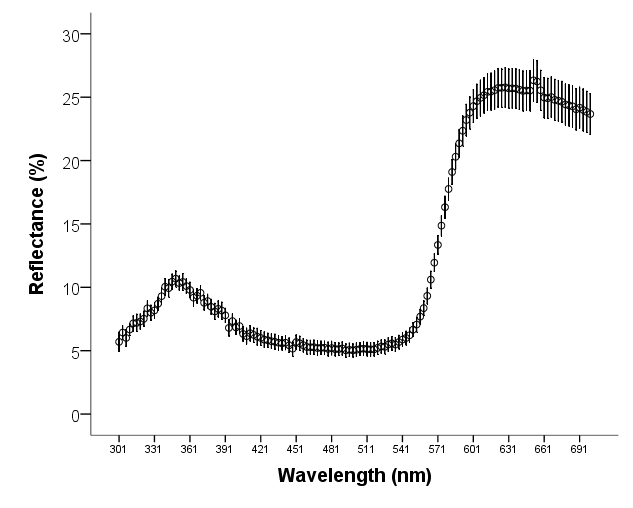


**Supporting Figure 2. Color spectrum of the eye ring of red-legged partridges.** Bars represent means ± SE. *n* = 20.

In addition, we have previously correlated the redness measurement (i.e. red chroma) obtained from our Avantes DH-2000 spectrometer (i.e. calculated as 600-700 nm reflectance divided by 300-700 nm reflectance) and our picture-based variable, which was assessed in the same point where the spectrometer probe was placed to avoid the effect of surface heterogeneity. We detected a significant and positive co-variation (Lessells & Boag 1987; *r* = 0.86, *p* = 0.001, *n* = 30). Similar significant correlations (though poorer) have been found in carotenoid-based traits of other similar species (red grouse; Mougeot et al 2007 Funct Ecol 21: 886–898). Note that the alternative measurement of redness by spectrometry (i.e. the red hue: wavelength of maximum slope) is independent from variability within the UV range. Moreover, in another study about the red-legged partridge coloration, circulating carotenoid levels were not correlated to UV chroma (*p* = 0.20) in bare parts, but positively correlated to UV-independent redness (*p* < 0.001; table 1 in Perez-Rodriguez 2008 Behav Ecol Sociobiol 62: 995-1005).

Here we also list other details of our photographic technique. We used an Olympus e-500 reflex digital body with an objective Olympus Zuiko Digital 14-45mm (1:3.5-5.6) fixed at 25mm. The aperture size, exposure time and other parameters of the camera were always the same: F 10, 1/13s, 100-ISO and manual focus. The distance between the objective and the bird was fixed at 35.5 cm by means of the above cited Repro Base (model RS3 XA; Kaiser Fototechnik, Buchen). No flash was used. The Repro Base included a system with two lights RB260 2x11W 6000ºK (Kaiser Fototechnik) also placed at 35.5cm from the bird’s head. The base included a grid board that allowed placing the birds always in the same position.

Color was measured on pictures using Adobe Photoshop. The redness of the upper and lower mandible and the eye ring was determined by recording mean values of red, green and blue components (RGB system) for each area. The pictures used in this study only included the head of the bird. Thus, the images were different from those used to analyze melanin-based color in Galvan & Alonso-Alvarez (2009, Proc R Soc 276: 3089–3097). One picture per bird was obtained. Previous work on duplicate photographs (moving the bird and again placing it under the camera) reported very high repeatabilities (*r* > 0.90, for each trait; 48 birds, 96 pictures; see also SI). Moreover, mean RGB values obtained per duplicate for each area in the same picture were repeatable (all *r-*values > 0.90, *P* < 0.001, *n* = 71), average values being used. Analyses in Photoshop were performed by the same person (i.e., Maria Ester Ferrero; research technician) who was blind to the bird’s identity.

Values of hue in the eye ring, upper and lower mandibles in our experimental birds (diquat or α-MSH-treated birds) were within the natural range after comparing them with a sample of 50 wild birds (experimental *vs.* wild birds’ range hue values: eye ring: 2.50º-12.49º *vs.* 2.16º-12.76º; upper mandible: 2.01º-9.49º *vs.* 2.53º-9.89º; lower mandible: 1.25º- 7.99º *vs.* 2.00º-8.30º).

**Measurement of plumage melanization**

One photograph of the breast and another photograph of the left flank of birds were taken with the same digital camera (Olympus E-500) at the same age that the head was photographed (i.e. 93 days of age). The birds were held in the same posture and at a fixed distance from the camera (same parameters above), but the focus here was broader (i.e., 14 mm). The black and brown areas were measured with the ‘magic wand’ tool of Adobe Photoshop CS. To control for a subtle change in technical parameters of the camera between the two incubation series of birds, all measurements were standardized (mean = 0, SD = 1). The repeatability of the measurements taken twice was very high (*r* = 0.93, *F*_72,73_ = 27.74; *r* = 0.89, *F*_70,71_ = 18.04; and *r* = 0.93, *F*_70,71_ = 108.02, for black spotted bib, eumelanic and pheomelanic flank bands, respectively; all *P* < 0.0001), and therefore, mean values were used (also Galvan & Alonso-Alvarez 2009 Proc R Soc B 276: 3089–3097).

Values of surface for black spotted bib and black and brown flank bands in our experimental birds (diquat or MSH-treated birds) were within the natural range after comparing them with a sample of 50 wild birds (experimental vs wild birds’ range in pixels: black spotted bib: 25655-80599 *vs.* 12695-81785; black flank bands: 12938-30787 *vs.* 5072-32816 ; brown flank bands: 12308-55702 *vs.* 9322-96047),

**Saturated models before the backward stepwise procedure**

**Supporting Table 1.** Generalized linear mixed model testing the influence of the experimental treatments and sex on red intensity (PC1) of developing red-legged partridges.

|  | *F* | d.f. | *P*-value |
| --- | --- | --- | --- |
| diquat | 3.80 | 1,22.9 | 0.064 |
| α-MSH | 0.01 | 1,44.3 | 0.954 |
| sex | 0.42 | 1,45.5 | 0.522 |
| diquat x α-MSH | 1.42 | 1,49.8 | 0.238 |
| diquat x sex | 3.72 | 1,50.3 | 0.060 |
| α-MSH x sex | 0.01 | 1,51.9 | 0.937 |
| diquat x α-MSH x sex | 0.66 | 1,49.8 | 0.419 |
| red chip | 267 | 1,39.4 | 0.001 |
| body mass change | 2.22 | 28.9 | 0.147 |
|  |  |  |  |
| random factors | *Z* |  | *P*-value |
| parental identity | 1.86 |  | 0.031 |
| aviary | 0.27 |  | 0.392 |

**Supporting Table 2.** Model testing the influence of the experimental treatments and sex on the change in circulating carotenoid levels.

|  | *F* | d.f. | *P*-value |
| --- | --- | --- | --- |
| diquat | 5.09 | 1,28.2 | 0.032 |
| α-MSH | 5.98 | 1,54.4 | 0.018 |
| sex | 0.19 | 1,57.9 | 0.663 |
| diquat x α-MSH | 0.06 | 1,55.7 | 0.812 |
| diquat x sex | 0.12 | 1,56.8 | 0.726 |
| α-MSH x sex | 0.18 | 1,57.3 | 0.675 |
| diquat x α-MSH x sex | 0.40 | 1,58.2 | 0.531 |
| pre-treatment carotenoid level | 65.4 | 1,25.1 | < 0.001 |
| body mass change | 9.64 | 1,49 | 0.003 |
|  |  |  |  |
| random factors | *Z* |  | *P*-value |
| parents’ identity | 0.24 |  | 0.407 |
| aviary | 0.78 |  | 0.219 |

**Supporting Table 3.** Model testing the influence of the experimental treatments and sex on the change in circulating cholesterol levels during the experiment.

|  | *F* | d.f. | *P*-value |
| --- | --- | --- | --- |
| diquat | 2.23 | 1,48.5 | 0.142 |
| α-MSH | 1.68 | 1,42 | 0.202 |
| sex | 2.61 | 1,39.3 | 0.114 |
| diquat x α-MSH | 0.94 | 1,48.6 | 0.338 |
| diquat x sex | 2.46 | 1,47.7 | 0.124 |
| α-MSH x sex | 0.20 | 1,56.2 | 0.654 |
| diquat x α-MSH x sex | 0.03 | 1,45.5 | 0.854 |
| pre-treatment cholesterol level | 28.8 | 1,49.4 | < 0.001 |
| Body mass change | 0.10 | 1,46.8 | 0.754 |
|  |  |  |  |
| random factors | *Z* |  | *P*-value |
| parents’ identity | 0.82 |  | 0.206 |
| aviary | 1.04 |  | 0.148 |

**Supporting Table 4.** Model testing the influence of the experimental treatments and sex on the change in the level of circulating triglycerides during the experiment.

|  | *F* | d.f. | *P*-value |
| --- | --- | --- | --- |
| Diquat | 0.96 | 1,38.4 | 0.333 |
| α-MSH | 0.01 | 1,49.7 | 0.907 |
| Sex | 0.18 | 1,53.1 | 0.669 |
| diquat x α-MSH | 0.10 | 1,54.6 | 0.745 |
| diquat x sex | 2.09 | 1,53.5 | 0.154 |
| α-MSH x sex | 5.14 | 1,56 | 0.027 |
| diquat x α-MSH x sex | 0.26 | 1,56.7 | 0.613 |
| pre-treatment triglyceride level | 48.6 | 1,53.5 | < 0.001 |
| body mass change | 5.23 | 1,48.5 | 0.027 |
|  |  |  |  |
| random factors | *Z* |  | *P*-value |
| parents’ identity | 0.36 |  | 0.359 |
| Aviary | 0.98 |  | 0.163 |
